# Research on the Mechanism of In-Plane Vibration on Friction Reduction

**DOI:** 10.3390/ma10091015

**Published:** 2017-09-01

**Authors:** Peng Wang, Hongjian Ni, Ruihe Wang, Weili Liu, Shuangfang Lu

**Affiliations:** 1 Research Institute of Unconventional Oil & Gas and Renewable Energy, China University of Petroleum (East China), Qingdao 266580, China; wpainn@hotmail.com (P.W.); lushuangfang@upc.edu.cn (S.L.); 2 School of Petroleum Engineering, China University of Petroleum (East China), Qingdao 266580, China; wangrh@upc.edu.cn (R.W.); wp1020sy@163.com (W.L.)

**Keywords:** friction reduction mechanism, petroleum drilling, in-plane vibration, friction model

## Abstract

A modified model for predicting the friction force between drill-string and borehole wall under in-plane vibrations was developed. It was found that the frictional coefficient in sliding direction decreased significantly after applying in-plane vibration on the bottom specimen. The friction reduction is due to the direction change of friction force, elastic deformation of surface asperities and the change of frictional coefficient. Normal load, surface topography, vibration direction, velocity ratio and interfacial shear factor are the main influence factors of friction force in sliding direction. Lower driving force can be realized for a pair of determinate rubbing surfaces under constant normal load by setting the driving direction along the minimum arithmetic average attack angle direction, and applying intense longitudinal vibration on the rubbing pair. The modified model can significantly improve the accuracy in predicting frictional coefficient under vibrating conditions, especially under the condition of lower velocity ratio. The results provide a theoretical gist for friction reduction technology by vibrating drill-string, and provide a reference for determination of frictional coefficient during petroleum drilling process, which has great significance for realizing digitized and intelligent drilling.

## 1. Introduction

The phenomenon that vibration, including normal vibration and in-plane tangential vibration, can reduce the friction between rubbing pairs has been proven by many studies [1,2,3,4], and has been used to obtain lower friction in many engineering fields for many years, such as vibration compactors and plates, metal working, wire drawing, cutting, atomic force microscopes and so on [5]. In recent years, in order to reduce the friction force between drill-string and borehole to improve the transfer efficiency of weight on bit, petroleum-drilling engineering developed Agitator [6], Slider [7] and Xciter [8], which can excite drill-string vibrating axially, torsionally and laterally, respectively. Higher rate of penetration (ROP), improved tool-surface control, reduced drag and stick/slip are some of the benefits to reduce friction force along the drill-string through exciting the drill-string mildly while drilling directional wells in the process of petroleum exploitation. The drill-strings are generally thousands of meters, hence a higher proportion of drill-string can be excited under axial or torsional vibration than lateral vibration. Moreover, the lateral vibration of drill-string is more likely to cause fatigue rupture. Agitator and Slider technology based on axial vibration and torsional vibration have been widely used and have good application effect. However, there are difficulties in calculating frictional coefficient and friction force between drill-string and borehole wall, which will influence the design of construction plan. The materials of steel and rocks made by many research achievements cannot be applied directly. Research of the mechanism of friction reduction under in-plane vibrating and its influence factors cannot only guide relevant engineering application, but also can be used to explore the mechanism of friction [9].

Mechanism of friction reduction under vibrating conditions is the basis of engineering applications. In order to obtain lower friction force and better application effect, many scholars began to explore the mechanism of friction under vibration condition, and analyze its influence factors. At macro-scale, the friction reduction mechanism of normal vibration [10,11] is that vibrations make the distance and normal contact force changes periodically. The friction reduction mechanism of in-plane vibration is that in-plane vibration changes the direction of friction which leads to the reduction of average friction force in a vibration cycle. Friction reduction can be obtained only when the amplitude of vibration velocity is larger than sliding velocity for longitudinal vibration [12,13,14,15,16,17,18] and only when the angle between the directions of vibration velocity and sliding velocity is more than zero for transverse vibration which will make the friction in sliding direction disassembled [14,15,16,17,18].

Above-mentioned mechanisms of friction reduction under vibration conditions at macro-scale explain some experimental phenomena to some extent. However, the effect of friction reduction under vibrations changes significantly. Mainly because friction force is not only concerned with amplitude, frequency and direction of vibration, but also with the relative sliding velocity and surface properties of rubbing pairs [19]. However, the mechanisms of friction reduction under vibrating conditions are still not very clear although a lot of research has been carried out. One of the reasons is that the Coulomb friction model adopted in the above analysis did not take into account the deformation of contact area in the process of sliding, especially while the friction under vibrating conditions is in a state of “stiction-sliding-stiction” which makes the deformation of contact area non-negligible. Therefore, a large number of scholars adopted dynamic friction model, including Dahl model [19,20], LuGre model [21], Dupont model [22,23] to analyze the effect of deformation of contact area on the change of friction during in-plane vibration, which obtained better simulation results [24,25,26,27].

From the above analysis we can see that the friction models only consider the global property of contact surfaces, and conduct unification hypothesis for the distribution and deformation of surface asperities, and attribute all influences of factors to an integrated coefficient. The micro-topography of surface is not considered. Although the models established have unified form for different rubbing pairs, accurate acquiring of the key parameters in the models is difficult. For all these reasons, the calculation results for different rubbing pairs are quite different. In essence, the above research is a correction of the friction coefficient, and does not refer to the influences of vibration on the friction coefficient itself. It is easy to understand the decrease of friction coefficient caused by the periodical change of distance and normal force between surfaces under normal vibration conditions, however, research on how the in-plane vibration influences the frictional coefficient has not been reported. Therefore, it is strongly necessary to study how the in-plane vibration influences the friction coefficient and provides guidance for its application in engineering fields.

In this paper, materials of alloyed steel and rocks are adopted to research the mechanism of in-plane vibration on friction reduction in petroleum exploitation. First, the friction experiment between drill-string material (i.e., alloyed steel) and rocks is carried out at different amplitudes, frequency and directions of in-plane vibration. Second, a deterministic friction model is established to analyze the effect of in-plane vibration on frictional coefficient and friction force in sliding direction. Third, the computed results of already existing friction models and the model modified in this paper are compared with experimental results. It was found that the frictional coefficient in sliding direction decreased significantly after applying in-plane vibration on the bottom specimen. The modified model can improve the accuracy to some extent in predicting frictional coefficient under vibrating conditions, especially under the conditions of lower velocity ratio.

## 2. Experiment

### 2.1. Experimental Setup

**Figure 1:**
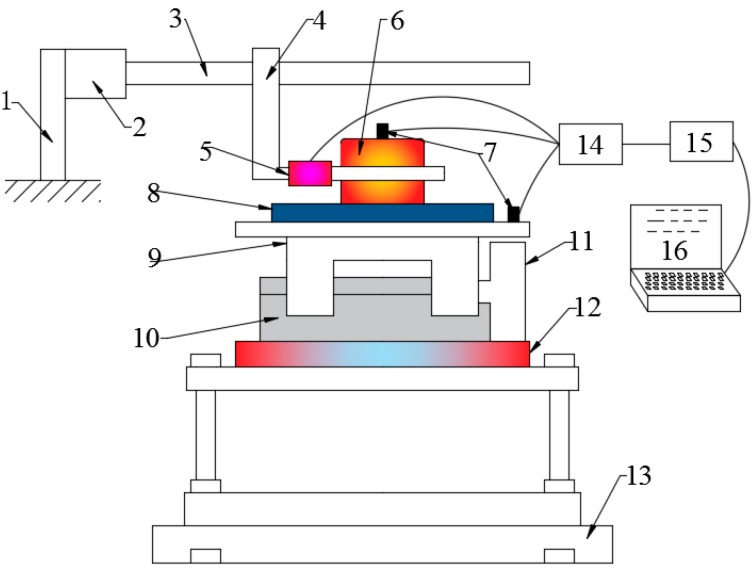
Schematic diagram of setup. **1**. Driving arm holder; **2**. linear motor; **3**. screw rod; **4**. driving arm; **5**. load cell; **6**. upper specimen; **7**. accelerometer; **8**. bottom specimen; **9**. sliding table; **10**. slide rail; **11**. electromagnetic modal exciter; **12**. movable disc; **13**. base plate; **14**. amplifier; **15**. A/D converter; **16**. PC.

The schematic diagram of experiment set-up is shown in Figure 1. Driving arm drives the upper specimen moving forward at a constant speed $V_{c}$. Electromagnetic modal exciter drives the sliding table together with the bottom specimen vibrating back and forth at a cosinoidal speed $V_{v}$. The direction of vibration speed $V_{v}$ can be adjusted by a rotating movable disc. Friction force in the sliding direction between upper and bottom specimens and the acceleration of specimens under different vibrating parameters and directions can be measured using this setup. For more details about the setup and the calculation process of frictional coefficient refer to reference [28].

### 2.2. Materials

**Figure 2:**
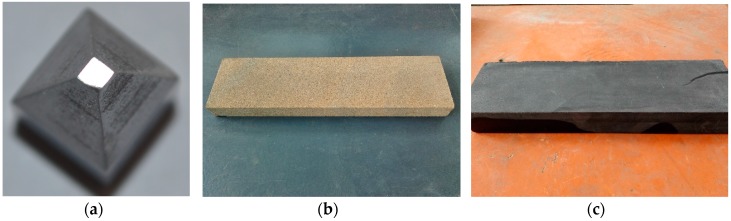
Specimens. (**a**) Alloyed steel; (**b**) sandstone; (**c**) shale.

**Figure 3:**
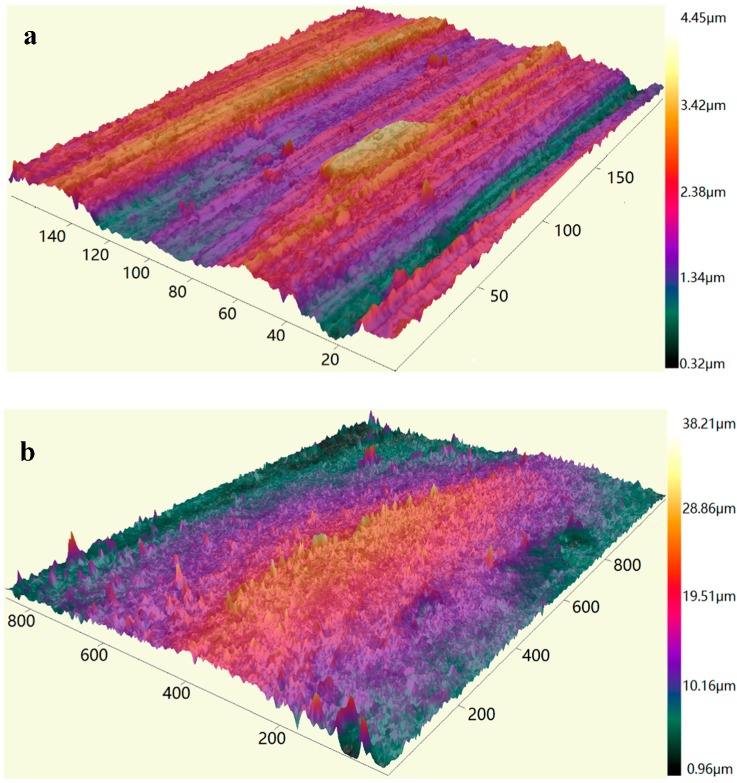

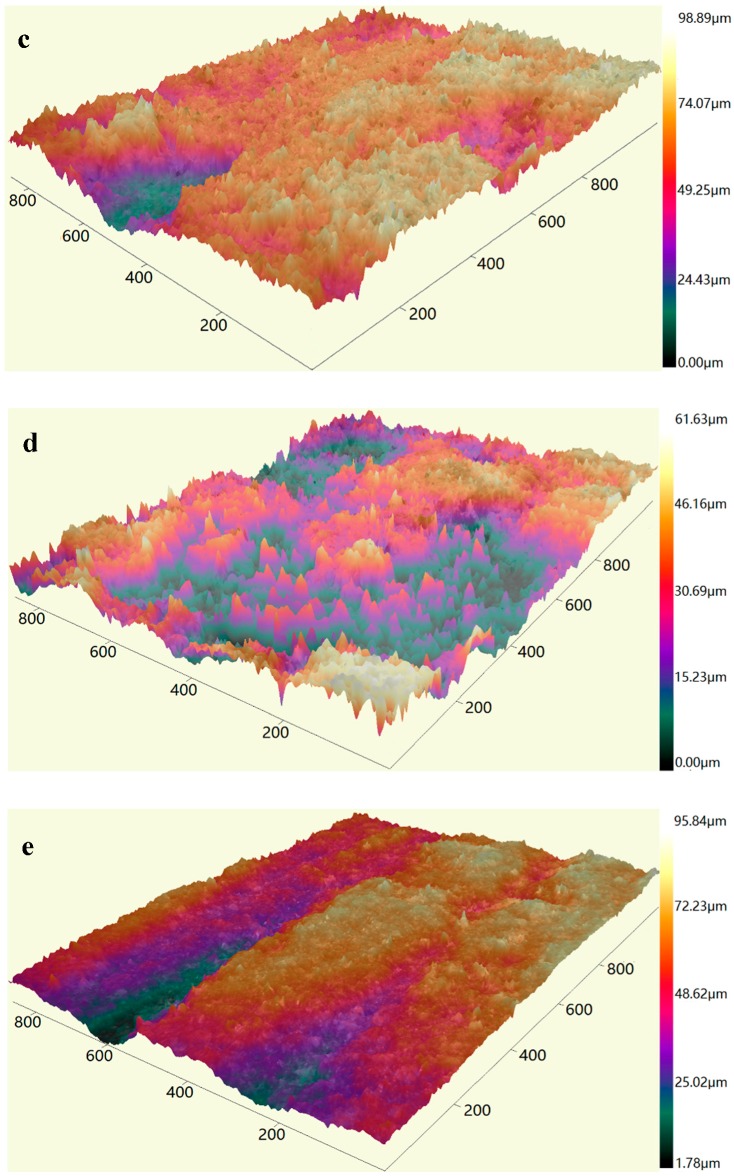

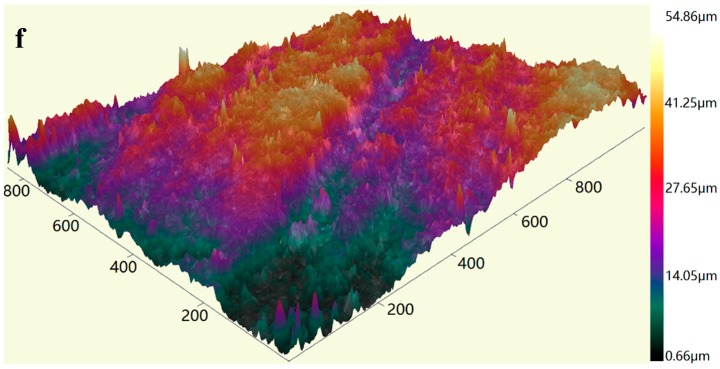
3-D measurement of specimens. (**a**,**b**) Before and after test of alloyed steel; (**c**,**d**) before and after test of sandstone; (**e**,**f**) before and after test of shale.

In petroleum drilling engineering, the drill-string is made of alloyed steel with steel grade D, E, X, G, S, the joint of drill-string is often made of 35CrMo with higher steel grade. 35CrMo was adopted as the material of upper specimen in this study. Three kinds of material were chosen as the borehole materials, including 35CrMo, sandstone and shale (see Figure 2). The specimens were finished by sandpaper 400 under a normal force of 10 N during 20 s. The average roughness of the 35CrMo (upper specimen) test sample was found to be 0.83 μm (RMS). The average roughness of the 35CrMo, sandstone and shale (bottom specimens) test specimens were found to be 1.20, 5.38 and 4.26 μm (RMS), respectively. ‘Zeta-20 metrology systems’ was used to scan the surface topography of rubbing pairs. The sampling interval, measurement area and type of filtering are listed in Table 1. The main mechanical characteristics of specimens are shown in Table 2. The 3-D shapes of specimens before and after experiment are shown in Figure 3.

### 2.3. Procedures

**Figure 4:**
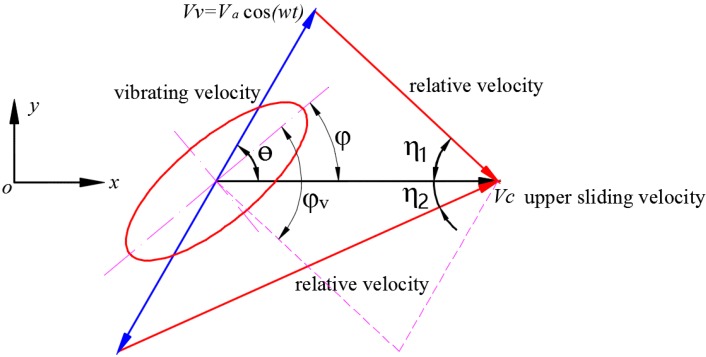
The direction of in-plane vibration and its effect on orientation angle.

The relation between sliding direction, vibrating direction and relative velocity direction in the condition of in-plane vibrations is shown in Figure 4. Three series of tests were conducted by changing the exciting force, frequency (ω) and vibrating direction (θ) of in-plane vibration, respectively, to study the effect of these factors on friction force and frictional coefficient in the sliding direction. The upper specimen slides along the *ox* direction with a consistent velocity of $V_{c}=1.07$ mm/s. The bottom specimen vibrates with a cosinoidal velocity $V_{v}=V_{a}\cos\left(\omega t\right)$ in the plane of *xoy*, the angle between sliding direction and vibrating direction θ is respectively set to 0°, 30°, 60° and 90°. The amplitude of vibrating velocity $V_{a}$ was calculated using the acceleration of specimens and changed by adjusting the exciting force and frequency. The nominal normal force were pre-set to be $p_{nom}=$ 13.5 MPa and kept constant during the experiments. All the tests are conducted at room temperature (between 25 and 30 °C) and moderate relative humidity (between 40% and 60%). Each test was repeated over three times under the same conditions to assure the reliability of the experimental data.

### 2.4. Results and Discussion

**Figure 5:**
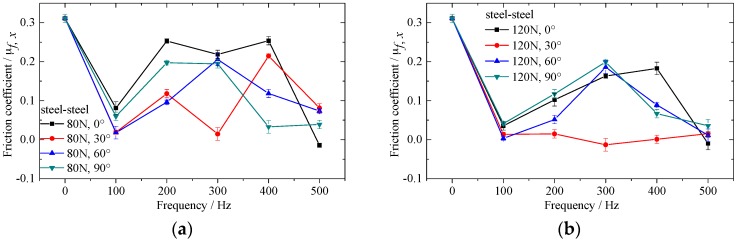

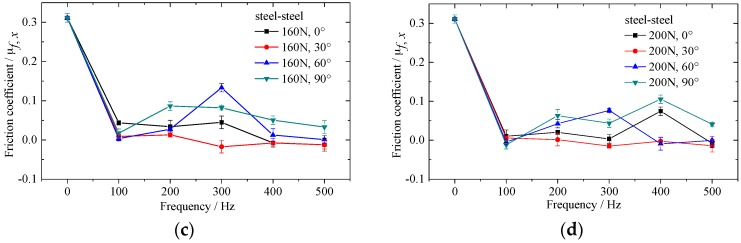
Effect of in-plane vibration on frictional coefficient between alloyed steel and steel. (**a**) Exciting force equals to 80N; (**b**) exciting force equals to 120N; (**c**) exciting force equals to 160N and (**d**) exciting force equals to 200N.

**Figure 6:**
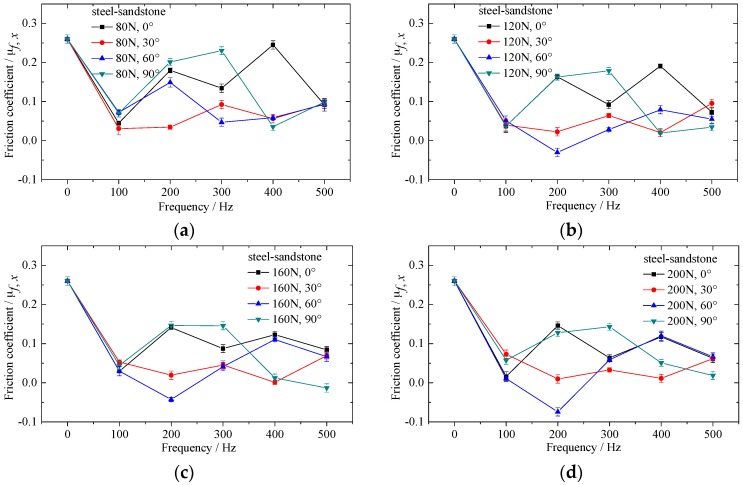
Effect of in-plane vibration on frictional coefficient between alloyed steel and sandstone. (**a**) Exciting force equals to 80N; (**b**) exciting force equals to 120N; (**c**) exciting force equals to 160N and (**d**) exciting force equals to 200N.

**Figure 7:**
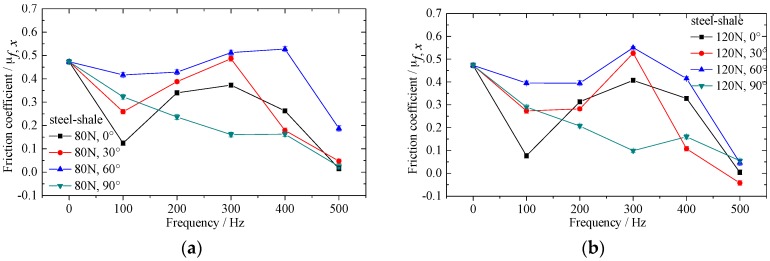

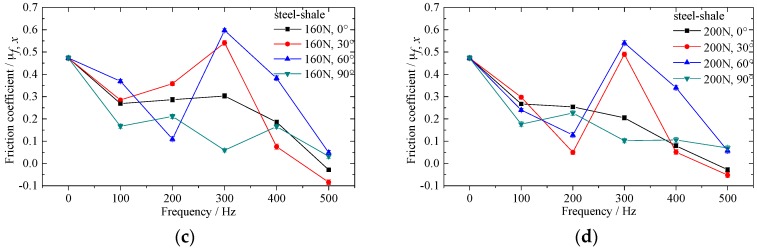
Effect of in-plane vibration on frictional coefficient between alloyed steel and shale. (**a**) Exciting force equals to 80N; (**b**) exciting force equals to 120N; (**c**) exciting force equals to 160N and (**d**) exciting force equals to 200N.

Figure 5, Figure 6 and Figure 7 show the effect of vibrating parameters and directions on frictional coefficient for three kinds of rubbing pairs, respectively. From these figures, the frictional coefficient decreases evidently when the in-plane vibrations are applied on the bottom specimens. The frictional coefficient decreases as the exciting force increases, and fluctuates wildly as the exciting frequency increases. Compared with steel and sandstone, the frictional coefficient between steel and shale shows lower friction reduction efficiency and severer fluctuation.

Meanwhile, the vibrating directions have huge impact on the frictional coefficient in the sliding direction. The frictional coefficient between steel and steel is smaller when the angle θ equals to 30°. The frictional coefficient between steel and sandstone is smaller when the angle θ equals to 30° and 60°. However, the frictional coefficient between steel and shale shows no clear regularity under the conditions of different vibrating directions.

The effect of vibrating force and frequency on frictional coefficient can attribute to the change of amplitude of vibration velocity and normal vibration induced by in-plane vibration [28]. The influence of vibrating directions on frictional coefficient for different rubbing pairs is inconsistent, which illustrates that the property of bottom specimens also influences the frictional coefficient to a great extent under the same motion and vibrating parameters. Therefore, the microcosmic properties of specimens should be considered in the study of friction reduction mechanism under vibrating conditions.

## 3. Theoretical Model

In the engineering application of friction reduction by vibrating, more attention is paid to the friction force reduction in a specific direction, for example, the axial direction in petroleum drilling. The direction change of friction force induced by in-plane vibration is usually thought to be the main reason for friction reduction under vibration conditions. While in fact, not only the direction of friction force but also the frictional coefficient will change during in-plane vibration process. In this section, existing friction calculation model under in-plane condition is modified by considering the effect of in-plane vibration on friction force and frictional coefficient simultaneously.

### 3.1. Contact Model under In-Plane Vibration

**Figure 8:**
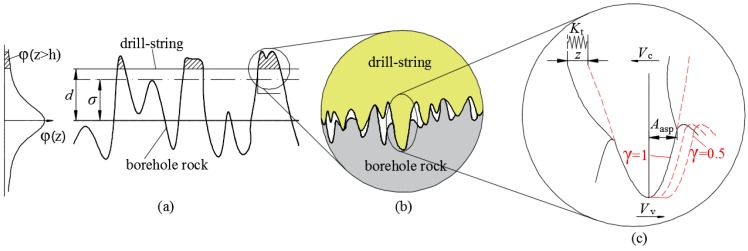
Real contact area on macroscopic scales. (**a**) Equivalent contact of drill-string and borehole wall; (**b**) asperities contact on micro-scale; (**c**) deformation of a single asperity.

Real contact happens only in partial asperities of surfaces when the drill-string contacts the borehole wall. Assuming the root mean square of surface height of drill-string and borehole wall equal to $\sigma_{1}$ and $\sigma_{2}$, respectively. The separation of their mean planes at a normal force equals to *d*. Based on tribology theory, considering the surface of drill-string is smoother than the surface of borehole wall, the contact between drill-string and borehole wall can be converted to a smooth surface and a rough surface whose root of mean square equals to $\sigma =\sqrt{\sigma_{1}^{2}+\sigma_{2}^{2}}$, as shown in Figure 8a.

In the contact model of actual rough surfaces, the relationship of real contact area and load actually depends on the contact status and distribution of asperity height. When the asperities are in plastic contact state, the real contact area always keeps linear relationship with load no matter the height distribution of surfaces. When the asperities are in elastic contact state, the real contact area also can be thought to be linear with load, while the coefficient is a little different under different height distribution of surface asperities [29]. Experimental results of Pullen and Williamson [30] indicate that if the indented softer material is restricted by rigid boundary and nominal contact pressure $p_{nom}>0.3H$, the linear relationship between real contact area and load is no longer true because of the lifting of non-contact region of softer material resulting from volume conservation. In the borehole environment, borehole wall is surrounded and restrained by surrounding rocks which satisfy the limiting condition of volume conservation model. Hence, the fraction of real contact area can be expressed as follows [31]:

(1) $$\alpha =\frac{p_{nom}}{H+p_{nom}}$$

where α is fraction of real contact area, i.e., degree of contact, dimensionless; $p_{nom}$ is nominal contact pressure, Pa; *H* is the hardness of the soft material, Pa. 

However, the relatively smooth surface is actually rough on a smaller scale. Asperities with larger height contact first, part of the contact asperities of harder surface penetrate the softer surface (see Figure 8b) until the real contact area reaches a certain value to realize equilibrium of forces in normal direction. In case of tangential relative motion, the contact area between contact asperities will change, and the part exposed to the softer surface of these contact asperities of harder surface will generate deformation *z* (see Figure 8c). The fraction of real contact area depends on elasticity resuming performance of rough surface:

(2) $$\alpha =\gamma \cdot \int_{d}^{\infty }\varphi \left(z\right)dz$$

where *d* is separation of mean planes of a contact pairs, m; γ is the elastic recovery factor, γ=0.5 for fully plastic materials, γ=1 for fully elastic materials; φ(z) is the surface height probability density distribution.

From the above, inserting Equation (2) into Equation (1) we can obtain:

(3) $$d={\mathrm{erfc}}^{-1}\left(\frac{2}{\gamma }\frac{p_{nom}}{H+p_{nom}}\right)$$

When in-plane vibration is applied to the bottom specimen, the fraction of real contact area will change more acutely, especially when the in-plane vibration is longitudinal. In order to keep force balance in normal direction the separation between surfaces *d* will change, which is one of the mechanisms that tangential motion arouses normal vibration. In this paper, the influence of in-plane vibration on fraction of real contact is not considered and the normal load remains constant.

### 3.2. Frictional Coefficient under In-Plane Vibration

Coulomb model and Dahl model have been widely accepted and used by most scholars for interpreting the mechanism of friction reduction under in-plane vibrations. Compared with Coulomb friction model, Dahl model not only considers the direction change of friction force but also the elastic deformation of asperities during vibrating process. Related studies [25,26,27] show that simulated results using Dhal model are more precise than Coulomb model because of the consideration of elastic deformation of surface asperities. However, Dahl model only considers the change of friction force with the offset of asperities, but does not consider the effect of in-plane vibrations on frictional coefficient itself. Therefore, we will take into account the effect of in-plane vibrations on the frictional coefficient in this section, and then replacing the dynamic frictional coefficient in Dahl model with the new frictional coefficient which will be discussed in this section.

**Figure 9:**
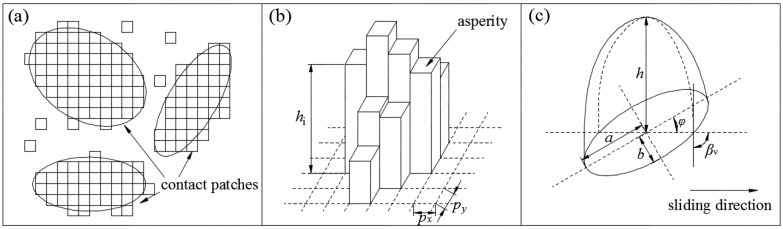
Structure of contact patches and an elliptical paraboloid [32]. (**a**) Contact patches in mean plane; (**b**) discrete asperities; (**c**) equivalent elliptical paraboloid.

Ma et al. [31] proposed a “deterministic contact patch model”. In this model, the contact asperities of specimens under high fractional real contact areas merge into larger ellipse contact patches. The part of these contact patches that penetrate the softer material are elliptical paraboloids, as is shown in Figure 9. Then, two contact specimens are transformed to a series of ellipses defined by the locations of their centroids, major and minor axis lengths, and the orientation angle φ. For the contact of drill-string and borehole rock, which is a combined elastic–plastic deformation system, deterministic contact patch model is also applicative.

The height of every elliptical paraboloid in Figure 9c can be calculated from the following formula:

(4) $$V_{ep}=\int_{0}^{h}\pi a^{\prime }\left(z\right)b^{\prime }\left(z\right)dz$$

(5) $$a^{\prime }\left(z\right)=a\sqrt{\frac{h-z}{h}}\cdot b^{\prime }\left(z\right)=b\sqrt{\frac{h-z}{h}}$$

(6) $$h=\frac{2V_{ep}}{A}=\frac{2}{n}\overset{n}{\underset{j=1}{\sum }}h_{j}=\frac{2}{n}\overset{n}{\underset{j=1}{\sum }}(z_{j}-d)$$

where $V_{ep}$ is the volume of elliptical paraboloid, m^3^; A is the area of base ellipse, m^2^; a and b are the major and minor semi axis of the base ellipse of paraboloid, respectively, m; h is the height of elliptical paraboloid, m; n is the discrete asperity number of contact patch; *d* is the separation of mean planes of contacting pairs, which can be calculated by Equation (3).

The acquisition of other parameters of an elliptical paraboloid, including *a*, *b* and *φ* can refer to reference [32]. These parameters of all contact patches can be determined by adopting above method.

Under the condition of in-plane vibrations, vibrating directions and ratio of vibrating velocity and sliding velocity will influence the orientation angle $\varphi_{v}$ and the attack angle $\beta_{v}$, and ultimately influence frictional coefficient $\mu_{v}$. If we exert in-plane vibration on a contact patch, the movement and force of it are shown in Figure 4. According to geometric relationships:

(7) $$\eta_{1}=\cos^{-1}\left(\frac{1-\kappa \cdot \cos\;\omega t\cdot \cos\left(\theta \right)}{\sqrt{{\left(\kappa \cdot \cos\omega t\right)}^{2}+1-2\kappa \cos\omega t\cos\left(\theta \right)}}\right)$$

(8) $$\eta_{2}=\cos^{-1}\left(\frac{1-\kappa \cdot \cos\;\omega t\cdot \cos\left(\pi -\theta \right)}{\sqrt{{\left(\kappa \cdot \cos\omega t\right)}^{2}+1-2\kappa \cos\omega t\cos\left(\pi -\theta \right)}}\right)$$

where θ is the angle between the directions of sliding velocity and vibration velocity, $0\leq \theta \leq \frac{\pi }{2}$, rad; $V_{a}$ is the amplitude of in-plane vibration velocity, m/s; $V_{c}$ is constant sliding velocity, m/s; velocity ratio $\kappa =\frac{V_{a}}{V_{c}}$.

The orientation angle between the sliding direction (i.e., *ox* axis) and the major axis of the ellipse under the condition of in-plane vibration can be expressed as:

(9) $$\varphi_{v}=\{\begin{array}{c} \varphi +\eta_{1},\;t=\left[0,\frac{T}{2}\right]\\ \varphi -\eta_{2},\;t=\left(\frac{T}{2},T\right] \end{array}$$

where φ and $\varphi_{v}$ are the orientation angle between the sliding direction (i.e., *ox* axis) and the major axis of the ellipse without and with in-plane vibration, rad.

The attack angle of the elliptical paraboloid under in-plane vibration was calculated according to the following formula [32,33]:

(10) $$\beta_{v}=\arctan\left(\frac{2h\sqrt{b^{2}\cos^{2}\varphi_{v}+a^{2}\sin^{2}\varphi_{v}}}{\chi ab}\right)$$

where $\beta_{v}$ is the attack angle of the elliptical paraboloid under in-plane vibration, rad; χ is the shape factor.

After obtaining the attack angle $\beta_{v\_j}$(j = 1, 2, …, m) of all contact patches in the representative area, the frictional coefficient generated by this area can be calculated by combining $\beta_{v\_j}$(j = 1, 2, …, m). The geometry of contact patches is determined by normal pressure, when the nominal normal pressure is not so high, the number of contact patches is huge, making it difficult for us to measure and calculate the attack angles of every contact patch. Considering that the arithmetic average attack angle of contact patches is independent of the nominal pressure except at very high pressure where all the contact patches eventually join together [31], the more meaningful measurement is arithmetic average attack angle of contact patches. The arithmetic average attack angle of contact patches under lower normal pressure can be obtained by measuring the arithmetic average attack angle of under relatively high normal pressure. The arithmetic average attack angle and frictional coefficient can be calculated by the following formula:

(11) $$\beta_{ave}=\frac{\sum_{j=1}^{m}\beta_{v\_j}\cdot A_{j}}{\alpha A_{nom}}$$

where $\beta_{ave}$ is the arithmetic average attack angle of all contact patches, rad.

The frictional coefficient of contact patches $\mu_{v}$ can be calculated according to Appendix A.

(12) $$\mu_{v}=\frac{F_{w}}{F_{n}}=\frac{\sum_{j=1}^{m}\mu_{v\_j}\left(\beta_{v\_j},f_{nk}\right)A_{j}H}{\sum_{j=1}^{m}A_{j}H}=\mu_{v}\left(\beta_{ave},f_{nk}\right)$$

The arithmetic average frictional coefficient of an in-plane vibration period can be obtained through integrating the instantaneous frictional coefficient of a period, which is expressed as:

(13) $$\mu_{ave}=\frac{1}{T}\int_{0}^{T}\mu_{v}\left(t\right)dt$$

where $\mu_{ave}$ is arithmetic average frictional coefficient during a in-plane vibration period; T is the period of in-plane vibration, s.

**Figure 10:**
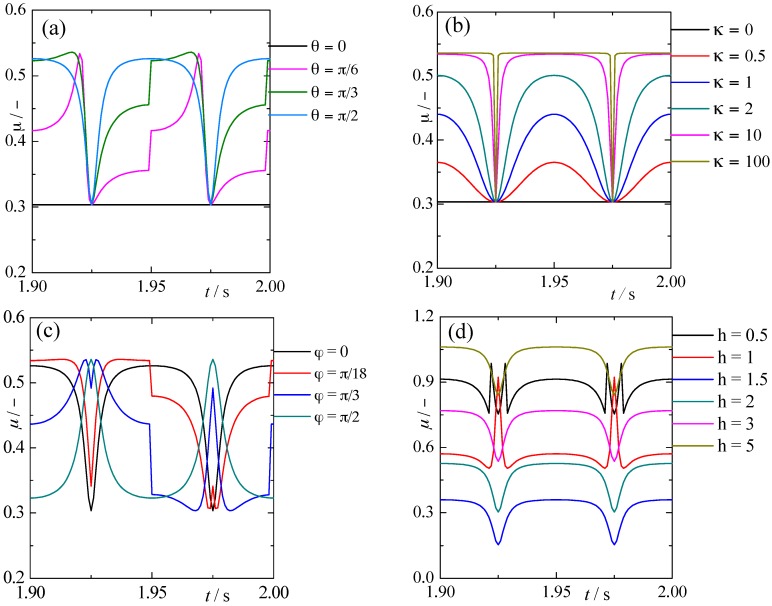
The effect of main parameters on frictional coefficient. (**a**) Vibration direction angle θ; (**b**) velocity ratio κ; (**c**) orientation angle of single contact patch φ; (**d**) height of single contact patch h.

Figure 10 shows the change of frictional coefficient of single contact patch during a vibration period and the influence of vibration direction angle θ, initial orientation angle of contact patch φ, velocity ratio κ and height of single contact patch h on frictional coefficient. If no special request, θ, φ, κ, h and $f_{nk}$ are equal to $\frac{\pi }{2}$, 0, 4, 2 and 0.83, respectively. From Figure 10a, the frictional coefficient remains constant during the whole period when θ=0, which is due to the fact that in-plane vibration has no influence on attack angle of contact patches in this condition. The frictional coefficient has different change rules with the increasing of θ when $0<\theta \leq \frac{\pi }{2}$, but one thing that they have in common is the maximum and minimum value of frictional coefficient at different θ are the same, which is due to the fact that the change of attack angle is inconsistent but the maximum and minimum value of attack angle are same for every θ. So the effect of vibration directions on frictional coefficient attribute to its influence on attack angle of contact patches. From Figure 10b, the frictional coefficient remains constant when velocity ratio κ=0, which is due to the fact that the direction of relative velocity is always consistent with the direction of sliding velocity and the attack angle is unchanged. The variation range of frictional coefficient expanded with the increasing of κ, and the frictional coefficient does not increase any more until the velocity ratio κ reaches a critical value. This phenomenon is because the maximum of attack angle in a period increases with the increasing of κ, which will make the frictional coefficient increase. From Figure 10c, the frictional coefficient has different variation tendencies with the increasing of orientation angle of contact patches from 0 to $\frac{\pi }{2}$, but the maximum and minimum value of frictional value are same for all orientation angles. This is also because the change rule of attack angle is inconsistent but the maximum and minimum value of attack angle are the same for every φ during a vibration period. From Figure 10d, the mean value of frictional coefficient decreases first and then increases with the increasing of height of the contact patch *h*. This phenomenon is because the frictional coefficient between surfaces experiences successive plowing, wedge-formation and cutting with the increase of *h* on the premise of $f_{nk}=0.83$. Therefore, there will be an appropriate attack angle $\beta_{opt}$ for every $f_{nk}>0.5$ can make the friction of two surfaces enter into wedge-formation region and obtain lower frictional coefficient. Besides, to which wear mode the contact belongs to depends on the topography and interfacial mechanics property of contact surfaces.

### 3.3. Frictional Force in Sliding Direction

After obtaining the frictional coefficient under condition of in-plane vibration $\mu_{v}$, we substitute it into Dahl model to calculate friction force in sliding direction. The Dahl’s model has the following form [19,20]:

(14) $$\{\begin{array}{c} \frac{dz}{dt}=V_{r}\cdot {\left[1-\frac{K_{t}}{\mu_{v}F_{n}}\cdot z\cdot sgn\left(V_{r}\right)\right]}^{i}\\ F_{f}=K_{t}\cdot z \end{array}$$

where $K_{t}$ is the tangential stiffness, N/m; i is a parameter that determines the shape of the stress-strain curve; *z* is the average deflection of asperities defined as the horizontal distance between the top and bottom points of asperity, m; $\mu_{v}$ is the kinetic friction coefficient under in-plane vibration; $F_{n}$ is the nominal normal force, N; $V_{r}$ is the relative velocity, m/s.

Tsai [25] improved Dahl model to make it not only suitable to longitudinal vibration but also in-plane vibration:

(15) $$\{\begin{array}{c} z\left(t+\Delta t\right)\approx z\left(t\right)+v\left(t\right)\left(1-\frac{K_{t}}{\mu_{v}F_{n}}\cdot z\left(t\right)\cdot sgn\left(v\left(t\right)\right)\right)\;i=1\\ z\left(t+\Delta t\right)=\{\begin{array}{c} \bar{T\left(t\right)P\left(t+\Delta t\right)}\;when\;\bar{T\left(t\right)P\left(t+\Delta t\right)}<z_{ss}\\ z_{ss}\;when\;\bar{T\left(t\right)P\left(t+\Delta t\right)}\geq z_{ss} \end{array}\;i=0 \end{array}$$

where P(t) and T(t) is the position of top and bottom point of asperities at time *t*, respectively, m; $\bar{T\left(t\right)P\left(t+\Delta t\right)}$ is the deflection displacement of top point and bottom point of asperities; v is the deflection velocity of asperities, m/s; $z_{ss}$ is the deflection displacement magnitude of asperities, m.

**Figure 11:**
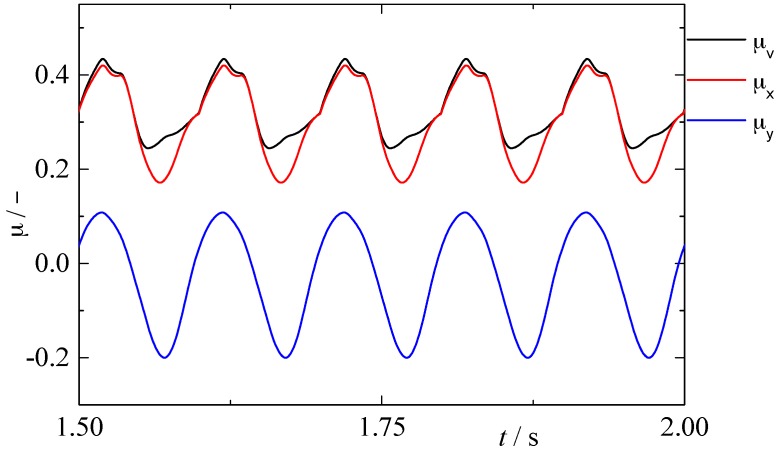
The change of frictional coefficient calculated by modified model ($\theta =\frac{\pi }{6}$, φ=0, κ=2, h=2 μm and $f_{nk}=0.83$, ω=62.8 (rad)).

So far, the modified model for calculating friction force under in-plane vibrations is established. Figure 11 shows the change of frictional coefficient in relative velocity direction, sliding direction (*x* axis) and perpendicular direction (*y* axis) with time. From Figure 11, frictional coefficient of different directions fluctuates acutely and periodically under the effect of in-plane vibration. The tendency of the frictional coefficient presented is the comprehensive result of the effect of in-plane vibration on friction direction and frictional coefficient, rather than only the influencing of the direction of friction force mentioned in related literature [25,26,27].

As can be seen from this section, many of contact patches formed under the action of normal and tangential load, then the orientation angle φ and attack angle β of contact patches in the sliding direction were determined. When in-plane vibration is applied to one of the specimens, this vibration may decrease the attack angle of some contact patches, meanwhile, increasing the attack angle of the other contact patches, which will decrease or increase the average attack angle of the surfaces, and the frictional coefficient are influenced at last. Meanwhile, the directions of friction force are also changed by the in-plane vibration. Only combining both of the above two effects, can friction force in sliding direction focused by us be judged as decreasing or increasing. It can be seen that the lowest frictional coefficient is obtained by choosing the sliding direction and in-plane vibration direction along the minimum average attack angle $\beta_{v}$ direction. Moreover, we can further decrease the frictional coefficient through increasing the velocity ratio κ and adjusting the height of asperities *h* to optimum. In the process of practical application, φ=0 usually corresponds to the machining direction. From this view, the frictional coefficient will be lower when the sliding occurs along the machining direction under the conditions of dry friction and boundary lubrication.

## 4. Experimental Verification of Numerical Result

**Figure 12:**
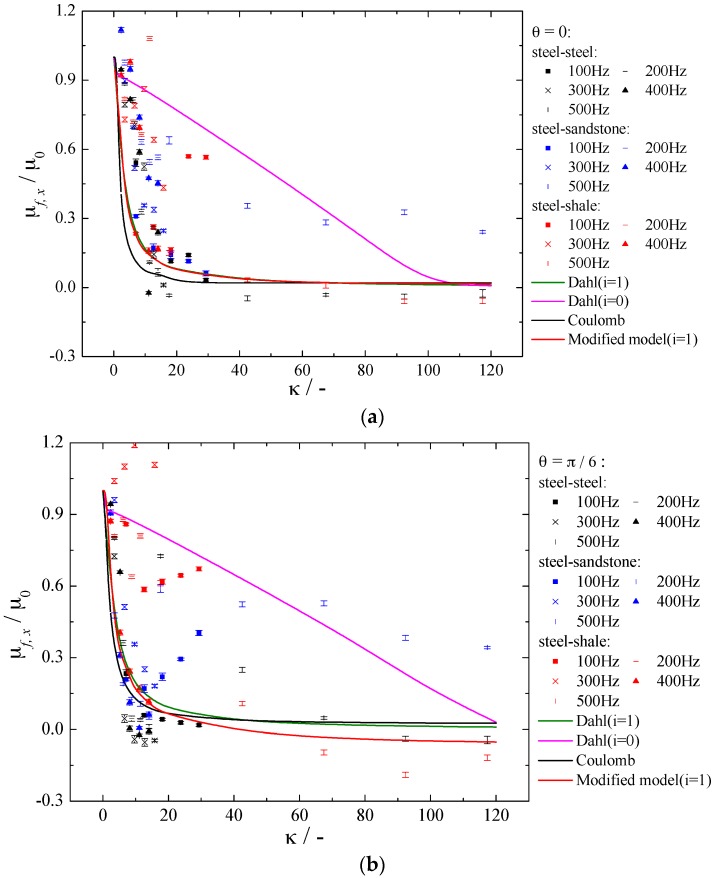

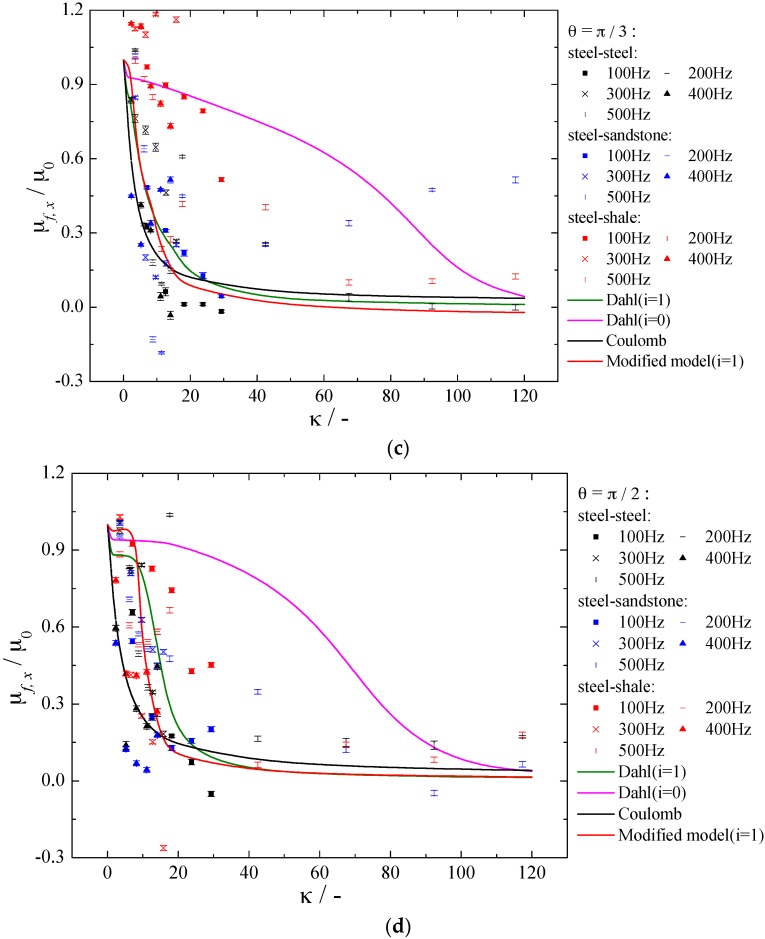
The effect of velocity ratio and vibration direction on friction coefficient ratio. (**a**) θ=0; (**b**) θ=π/6; (**c**) θ=π/3 and (**d**) θ=π/2.

In order to verify the capacity of modified model in predicting the frictional coefficient under in-plane vibration conditions, we compared the calculation results of Coulomb model, Dahl model and the modified model proposed in this paper with the experimental results in Section 2 (see Figure 12). The parameters used in models include $\varphi =\beta_{ave}=\frac{\pi }{4},f_{nk}=0.83,\;K_{t}=10^{5}\;N/m$. From Figure 12, the frictional coefficient ratio calculated by models of Coulomb and Dahl (*i* = 1) quickly decreases at first, and then reaches a plateau while the frictional coefficient ratio calculated by model of Dahl (*i* = 0) appears approximately linear decline with the increase of velocity ratio. Dahl model (*i* = 0) is less consistent with the experimental value than Dahl model (*i* = 1) because the offset of asperities calculated by Equation (15) when *i* = 0 is larger than *i* = 1, which will result in larger frictional coefficient. There is a critical value for κ. Dahl model (*i* = 1) has lower value than Coulomb model when κ is higher than this critical value. This critical values are 70, 45, 23, and 25 for θ=0, $\theta =\frac{\pi }{6}$, θ = $\frac{\pi }{3}$, and $\theta =\frac{\pi }{2}$, respectively. The results calculated by the modified model have similar tendency with Coulomb model and Dahl model (*i* = 1) in general. The calculation results of modified model and Dahl model (*i* = 1) are the same when θ=0. With the increasing of vibration direction angle, the calculated results of modified model are between Coulomb model and Dahl model (*i* = 1) under lower velocity ratio region, and are lower than results of Coulomb model and Dahl model (*i* = 1) under higher velocity ratio region. The consideration of orientation angle of contact patches in modified model make the frictional coefficient more sensitive to velocity ratio κ. Compared with alloyed steel, frictional coefficient of rock materials are harder to be predicted, especially when the applied in-plane vibration is intense.

**Figure 13:**
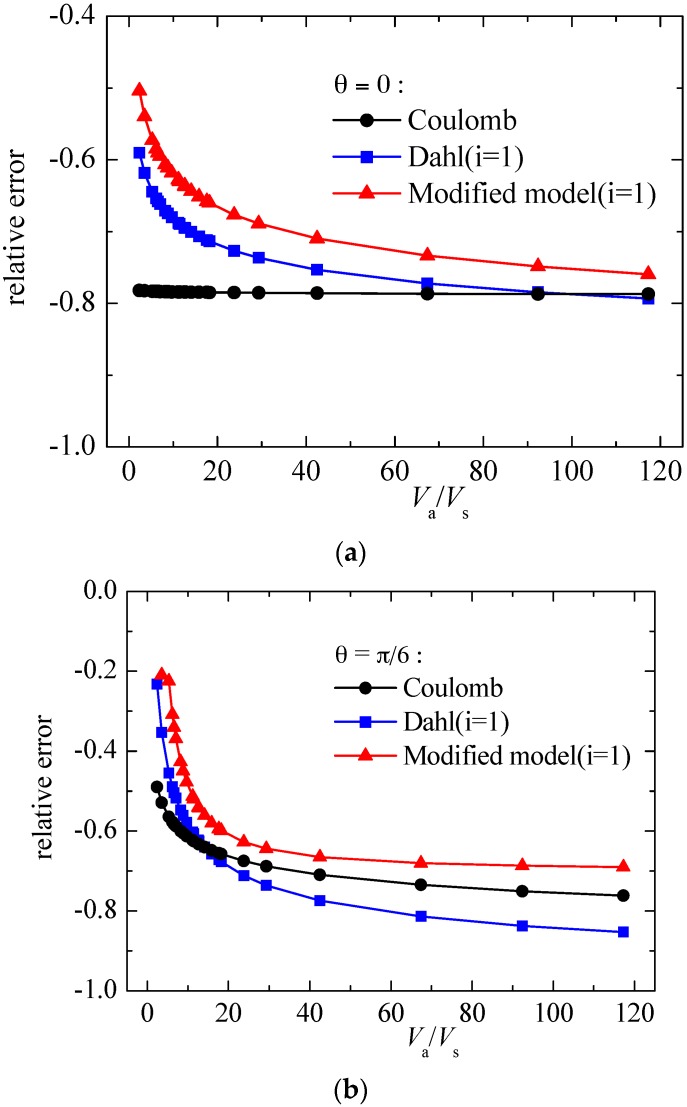

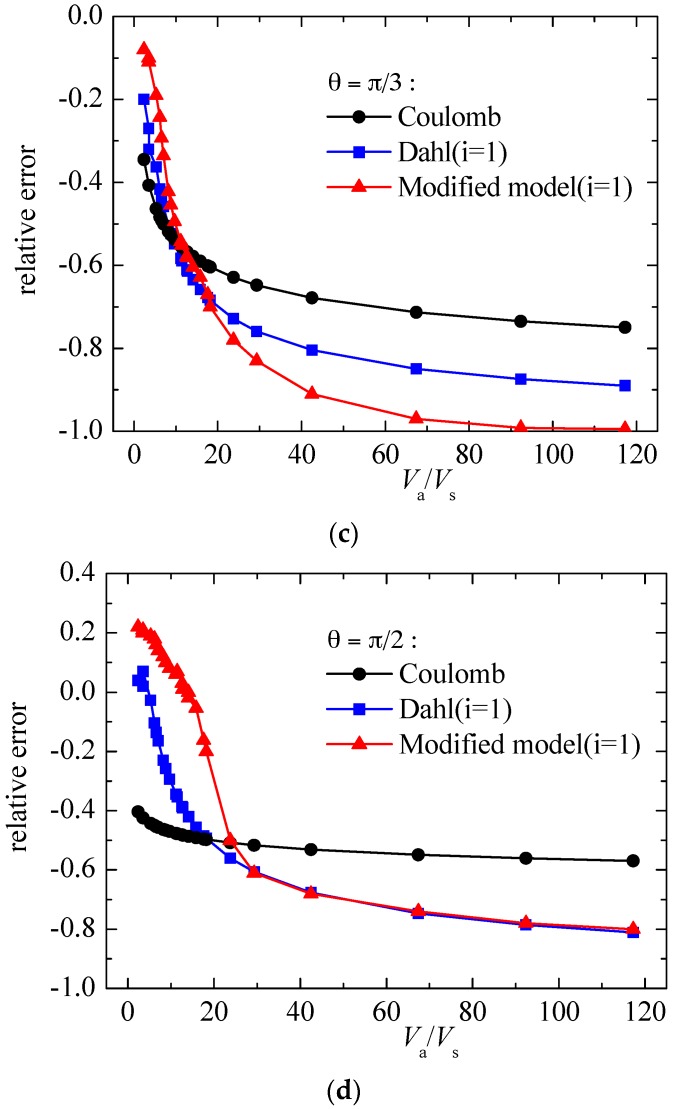
Relative error of modified model compared with Coulomb and Dahl models. (**a**) θ=0; (**b**) θ=π/6; (**c**) θ=π/3 and (**d**) θ=π/2.

To explain quantitatively the effectiveness of modified model proposed in this paper in predicting the friction coefficient, we calculated the relative errors of frictional coefficient between model calculating and experimental results (see Figure 13). As the calculating results of Dhal model (*i* = 0) are far from the experimental results, Dhal model (*i* = 0) is not compared in Figure 13. The relative error is defined as:

(16) $$RE=\frac{\mu_{f,x,m}-\mu_{f,x,e}}{\mu_{f,x,e}}$$

where *RE* is relative errors of frictional coefficient between model calculating and experimental results, dimensionless; $\mu_{f,x,m}$ is arithmetic average frictional coefficient in sliding direction calculated by models, dimensionless; $\mu_{f,x,e}$ is arithmetic average frictional coefficient in sliding direction measured by experiment.

From Figure 13, frictional coefficients calculated by all the three models are below the experimental results (except θ=π/2). For θ=0, the modified model can improve 3~58% in predicting the friction coefficient compared with Coulomb model, and 5~10% compared with Dahl model (*i* = 1). For θ=π/6, the modified model can improve 3~28% in predicting the friction coefficient compared with Coulomb model, and 1~16% compared with Dahl model (*i* = 1). For θ=π/3, the modified model can improve 0~27% in predicting the friction coefficient compared with Coulomb model, and 0~20% compared with Dahl model (*i* = 1) when velocity ratio $V_{a}/V_{s}\leq 10$, while reduce 0~30% when velocity ratio $V_{a}/V_{s}>10$. For θ=π/2, the modified model can improve 0~50% in predicting the friction coefficient compared with Coulomb model when velocity ratio $V_{a}/V_{s}\leq 24$, while reduce 0~25% when velocity ratio $V_{a}/V_{s}>24$, and the modified model can improve 0~40% in predicting the friction coefficient compared with Dahl (*i* = 1) model. From the above analysis we can see that the modified model can improve the accuracy to some extent in predicting frictional coefficient under vibrating conditions, especially under the conditions of lower velocity ratio.

## 5. Conclusions

A modified model is developed for predicting the frictional coefficient under in-plane vibration conditions. The reasons for friction reduction under in-plane vibrations are attributed to the direction change of friction force, tangential deformation of surface asperities and the change of frictional coefficient generated by the asperities of harder surface penetrating the softer surface. Vibration directions and velocity ratio influence the direction of relative velocity, which will indirectly result in change of frictional coefficient in direction of relative velocity. The change of relative velocity also influences the friction force in a certain direction. Surface topography and normal load jointly influence the formation and geometric parameters of contact patches. If we want to obtain lower driving force for a pair of determinate rubbing surfaces under constant normal load, the driving direction is better along the minimum arithmetic average attack angle direction, and applying intense longitudinal vibration on the rubbing pair. For petroleum drilling, the texture of borehole wall is along circumferential direction and perpendicular to the axial direction of drill-string because the rotational speed of drill-string is about one hundred times more than the axial speed of drill-string. Therefore, we can preliminarily speculate that the frictional coefficient of circumferential direction is lower than the frictional coefficient of axial direction, rather than having the same frictional coefficient in both the axial and circumferential directions. Theoretical and experimental research on the mechanism of in-plane vibration on friction reduction carried out in this paper can provide a theoretical gist for friction reduction technology by vibrating drill-string, and provide a reference for determination of frictional coefficient during petroleum drilling process, which has great significance for realizing digitized and intelligent drilling.

## Figures and Tables

**Table 1:** Information about measurement of surface.

Specimen	Measurement Area (μm)	Sampling Interval (μm)	Type of Filtering
a	232.4 × 174.4	0.4	median filter
b~f	1161 × 869.4	1.8	

**Table 2:** The main characteristics of the experimental material.

Material		Modulus of Elasticity (GPa)	Poisson’s Ratio	Hardness HB_30_	Roughness (RMS) after Surface Finishing (Sandpaper 400)	
					Before Test	After Test
Upper specimen	Alloyed steel	206	0.30	380	0.83	2.57
Bottom specimen	Alloyed steel				1.2	3.42
	Sandstone	40	0.25	226	5.38	4.28
	Shale	80	0.24	417	4.26	3.95

## Nomenclature

**Roman symbols**	
A	Area of base ellipse (m^2^)
a*,* b	Major and minor semi axis of the base ellipse of paraboloid (m)
$V_{c}$	Constant sliding velocity (m/s)
$V_{v}$	Instantaneous vibrating velocity (m/s)
$V_{a}$	Amplitude of in-plane vibration velocity (m/s)
$V_{r}$	The relative velocity (m/s)
v	The deflection velocity of asperities (m/s)
κ	Velocity ratio of $V_{a}$ and $V_{c}$
$p_{nom}$	Nominal normal pressure (Pa)
H	Hardness of the softer material (Pa)
*d*	The separation of mean planes of contacting pairs (m)
$K_{t}$	The tangential stiffness of surfaces (N/m)
*i*	Shape parameter of stress-strain curve
*z*	The average deflection of asperities (m)
$F_{n}$	Nominal normal force (N)
$F_{w}$	Tangential force (N)
P(t)	Position of top point of asperity at time t (m)
T(t)	Position of bottom point of asperity at time t (m)
$\bar{T\left(t\right)P\left(t+\Delta t\right)}$	Deflection displacement of top point and bottom point of asperity (m)
$z_{ss}$	Deflection displacement magnitude of asperities (m)
$V_{ep}$	Volume of representative elliptical paraboloid (m^3^)
h	Height of elliptical paraboloid (m)
m	Number of contact patches in a representative area (-)
n	Discrete surface points number of contact patch (-)
**Greek symbols**	
α	Fraction of real contact area, i.e., degree of contact (-)
ω	Circular frequency of in-plane vibration (rad/s)
θ	Angle between vibration direction and sliding direction, $0\leq \theta \leq \frac{\pi }{2}$ (rad)
φ *,* $\;\varphi_{v}$	Orientation angle between the sliding direction and the major axis of the ellipse without and with in-plane vibration,0≤φ,$\;\varphi_{v}\leq \frac{\pi }{2}$ (rad)
$\beta_{v}$	Attack angle of elliptical paraboloid under in-plane vibration (rad)
$\mu_{0}$	Kinetic friction coefficient (-)
$\mu_{v},\;\mu_{x},\mu_{y}$	Instantaneous frictional coefficient in relative direction, sliding direction and direction perpendicular to the sliding direction under in-plane vibration (-)
$\mu_{f,\;x}$	Arithmetic average frictional coefficient in sliding direction (-)
$\mu_{f,x,m}$	Arithmetic average frictional coefficient in sliding direction calculated by models (-)
$\mu_{f,x,e}$	Arithmetic average frictional coefficient in sliding direction measured by experiment (-)
$\mu_{ave}$	Arithmetic average frictional coefficient during a in-plane vibration period (-)
σ	Standard deviation of equivalent rough surface heights (m)
$\sigma_{1}$ *,* $\sigma_{2}$	Standard deviation of surface heights of upper specimen and bottom specimen (m)
γ	Elastic recovery factor, 0.5≤γ≤1 (-)
φ(z)	The surface height probability density distribution (-)

## Appendix A. Slip-Line Friction Model

Challen and Oxley [34] proposed a slip-line friction model based on a hard asperity plowing a softer surface under cutting, ploughing and wear conditions. The active mode can be determined by wear mode diagram [34,35]. The mode diagram describes the friction modes as a function of attack angle of a wedge-shaped asperity β and the interfacial shear factor $f_{nk}$, and the interfacial shear factor $f_{nk}$ can be calculated by the following equation:

(A1) $$f_{nk}=\frac{3\sqrt{3}\tau_{int}}{H}$$

The expressions of frictional coefficient in different regions are given by Challen and Oxley [34].

In the cutting regime:

(A2) $$\mu_{cutting}=\tan\left(\beta_{v}-\frac{\pi }{4}+\frac{1}{2}\cos^{-1}f_{nk}\right)$$

In the ploughing regime:

(A3) $$\mu_{ploughing}=\frac{A_{1}\sin\beta_{v}+\cos\left(\cos^{-1}f_{nk}-\beta_{v}\right)}{A_{1}\cos\beta_{v}+\sin\left(\cos^{-1}f_{nk}-\beta_{v}\right)}$$

(A4) $$A_{1}=1+\frac{\pi }{2}+\cos^{-1}f_{nk}-2\beta_{v}-2\sin^{-1}\left(\frac{\sin\beta_{v}}{\sqrt{1-f_{nk}}}\right)$$

In the wedge formation regime:

(A5) $$\mu_{wear}=\frac{\left(1-2\sin A_{2}+\sqrt{1-f_{nk}^{2}}\right)\sin\beta_{v}+f_{nk}\cos\beta_{v}}{\left(1-2\sin A_{2}+\sqrt{1-f_{nk}^{2}}\right)\cos\beta_{v}-f_{nk}\sin\beta_{v}}$$

(A6) $$A_{2}=\beta_{v}-\frac{\pi }{4}-\frac{1}{2}\cos^{-1}f_{nk}+\sin^{-1}\left(\frac{\sin\beta_{v}}{\sqrt{1-f_{nk}}}\right)$$
